# Cratonic basins as effective sediment barriers in continent-scale sediment routing systems of Paleozoic North America

**DOI:** 10.1038/s41598-023-37863-x

**Published:** 2023-07-10

**Authors:** Andrea L. Stevens Goddard, Olivia G. Thurston, David H. Malone, Patrick I. McLaughlin, Jack Stewart

**Affiliations:** 1 Department of Earth and Atmospheric Sciences, Indiana University, Bloomington, IN USA; 2 Department of Geography, Geology, and the Environment, Illinois State University, Normal, IL USA; 3 Illinois State Geological Survey, Champaign, IL USA

**Keywords:** Stratigraphy, Sedimentology, Tectonics

## Abstract

Provenance studies demonstrate the important control of plate boundary mountain building on continental sediment routing systems. Less well understood is if subsidence and uplift in cratons also has the potential to affect the organization of sediment routing systems on continental scales. New detrital zircon provenance data from the Michigan Basin in the Midcontinent of North America preserve evidence of intrabasin provenance heterogeneity in Cambrian, Ordovician, and middle Devonian strata. These results suggest that cratonic basins serve as effective sediment barriers that prevent mixing within and across basins from 10 to 100 s of millions of years. Internal sediment mixing, sorting, and dispersal may be achieved by a combination of sedimentary processes and inherited low relief topography. These observations are consistent with provenance data sets from eastern Laurentian Midcontinent basins that show locally and regionally variable provenance signatures during the early Paleozoic. By the late Devonian, provenance signatures throughout the basins homogenized, consistent with the emergence of transcontinental sediment transport systems associated with Appalachian orogenesis at the plate margin. These results demonstrate the importance of cratonic basins on local and regional sediment routing systems suggesting that these features may impede the integration of continental-scale sediment routings systems, particularly during periods of plate margin quiescence.

## Introduction

Sediment routing systems are a primary mechanism for redistributing Earth materials on continental crust. Numerous studies spanning space and time have documented the continental-scale integration of sediment routing systems during periods of tectonism that are controlled by high relief orogens at plate boundaries^1–7^. However, it is less clear if the geodynamics of continental interiors—including the depressions of the slowly subsiding cratonic basins themselves—can define local or regional drainage systems that have the potential to filter and/or disrupt continental scale sediment routing pathways. A cratonic basin sits on tectonically stable crust > 100 s of km from the nearest active plate boundary. Cratonic basins are generally long-lived (> 100 s of My), with slow, intermittent subsidence, and may be influenced by far-field effects of continental margin orogenesis, but are located far from high-topography sediment sources at plate margins that shed large volumes of clastic material^8^. Although initial subsidence in cratonic basins has been tied to supercontinent disassembly, subsidence persists for hundreds of millions of years through periods of both plate margin quiescence and compression^9–12^. Both their longevity and distance from orogenic sediment sources suggest that cratonic basins play an important role in transcontinental sediment mixing and transport patterns, but limited systematic work has established the effect of cratonic basins on sediment routing systems during periods of tectonic quiescence at plate margins. In the eastern Midcontinent of North America, Paleozoic strata within cratonic basins including the Michigan Basin, the Illinois Basin, and the Forest City Basin preserve provenance signatures that can be used to reconstruct sediment routing patterns within and between basins during periods of plate margin extension and quiescence followed by the growth of the Appalachian orogenic system^13^. Previous provenance analyses throughout the Midcontinent reconstruct Mississippian through Cretaceous transcontinental sediment routing systems evacuating materials from the Appalachians to the western margin of North America beginning during the Carboniferous—Permian Alleghenian orogeny and continuing through the Mesozoic^1,3–5^. These sediment routing systems are widely interpreted to have overwhelmed and traversed intermediate cratonic basins such as the Illinois, Michigan and Forest City Basins suggesting the presence of cratonic basins did not play a role in sediment organization during periods of plate boundary mountain building^5,14^.

However, the effect of cratonic basins, if any, on sediment routing systems during the early Paleozoic prior to these Appalachian orogenic events is mostly untested^15,16^ and sediment routing reconstructions interpret much of the Midcontinent as zones of sediment bypass interpolating hundreds of kilometers across entire basins. Clastic units including the Mt. Simon Sandstone (Cambrian) and the St. Peter Sandstone (Ordovician) found throughout most of the eastern half of North America have been widely interpreted as sheet sands that blanketed much of the continent, but the effects of cratonic basins or other intracontinental topography on sediment mixing and transport have been difficult to determine as these units are most commonly observed in the subsurface^17–19^.

This study presents new detrital zircon (DZ) data from Cambrian—Pennsylvanian strata in the Michigan Basin, a classic example of a cratonic basin^8,10^, including the first DZ data from Cambrian, Ordovician, and Devonian strata specifically interrogating the role of this cratonic basin on regional and continental sediment routing systems. Subsidence in the Michigan Basin initiated in the Cambrian and continued episodically throughout the Paleozoic producing alternating basin-centered and eastward-deepening subsidence patterns ^20–22^. The Basin overlies both the suture of the Yavapai and Mazatzal terranes^23^ and the Mesoproterozoic Midcontinent Rift that was inverted sometime during the Neoproterozoic at least by the Ediacaran^24–27^. Although the parts of the Midcontinent Rift may have been reactivated in the Paleozoic^27,28^, the crust underlying the Michigan Basin appears to have been tectonically inactive and sediment depositional patterns show no evidence of syndeformational sedimentation^22^. Cambrian—Mississippian strata were deposited in dominantly shallow marine environments. The geometry of the Paleozoic marine system largely followed the contours of the circular basin boundaries and marginal marine environments are recorded along the edges of the Michigan Basin^22,29–32^. It is difficult to predict the degree of mixing within the Michigan Basin given the basin geometry and marine environment alone. Based on modern-Pliocene observations in closed marine basins, we might expect along-shore transport and evacuation of sediment from the basin margins to the basin center during storms^33–35^, but there is no data constraining the degree of internal mixing or connectivity across the basin.

**Figure 1:**
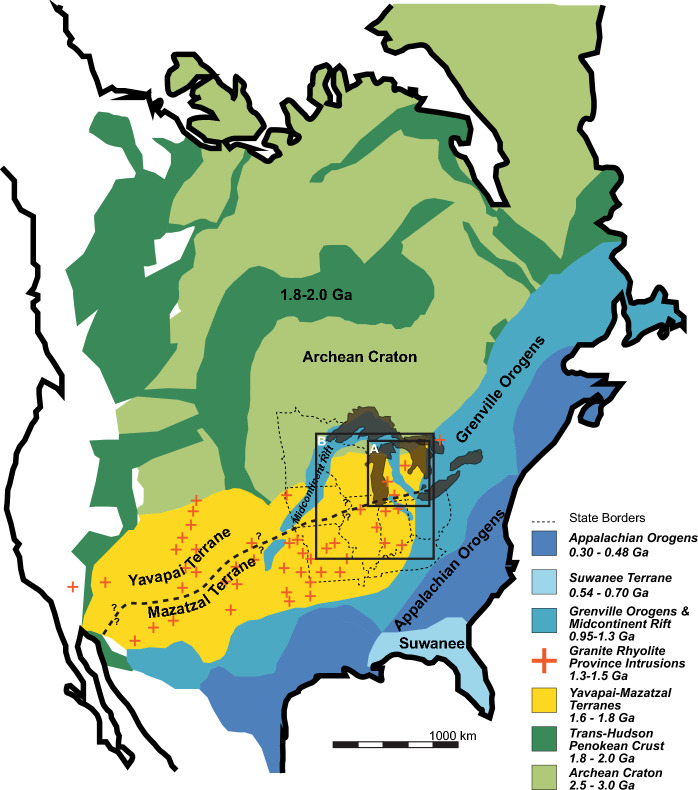
Basement source terranes of North America interpreted from Hoffman et al., 1989 and Gehrels & Pecha, 2014. Boxes show inset maps in Fig. 4 (Box A = Fig. 4A–D; Box B = Fig. 4E–H). Map was drawn in Adobe Illustrator 2020.

We compare provenance reconstructions within the Michigan Basin with a new regional compilation of detrital zircon data sets throughout the eastern Midcontinent of North America (Fig. 1). Our work provides evidence that the Michigan Basin served as a sediment trap for 100 + Myrs with little internal mixing across < 100 km scales. These observations suggest that cratonic basins can serve as effective sediment transport barriers for local, regional and potentially continental scale sediment distribution patterns, particularly during periods of tectonic quiescence at plate boundaries.

## Results and interpretation

### U–Pb ages of potential source areas

In Paleozoic Laurentia, seven crystalline basement and/or orogenic terranes with unique zircon U–Pb ages served as potential suppliers of Michigan Basin detritus (Fig. 1). All sources are represented within the Paleozoic stratigraphy of the Michigan Basin. These sources areas are summarized in Fig. 1 and include the Archean craton (3.0–2.5 Ga), Trans-Hudson/Penokean crust (2.0–1.8 Ga), the Yavapai-Mazatzal Province (1.8–1.6 Ga), Granite-Rhyolite Province intrusions (1.5–1.3 Ga), the Grenville orogen and rocks of the Midcontinent Rift (1.3–0.98 Ga), Suwanee Terrane (0.70–0.54 Ga), and the Appalachian orogen (0.48–0.3 Ma).

Sedimentary units with recycled material from these primary terranes may also serve as a source for Michigan Basin zircons. The Mesoproterozoic Baraboo quartzite west of the Michigan Basin preserves sandstones with either dominantly zircons associated Trans-Hudson/Penokean crust as well as lesser grains from the Archean craton or dominantly Yavapai-Mazatzal age zircons^7,36,37^. The Neoproterozoic Jacobsville Sandstone is preserved along the Midcontinent Rift north and northwest of the study area. The Jacobsville Sandstone has five distinct detrital zircon distributions that represent rift evolution with primary zircons from the Archean craton, Trans-Hudson/Penokean crust, Yavapai-Mazatazal Province, and the Grenville orogen^27,38^.

### Detrital U–Pb signatures in time and space

**Figure 2:**
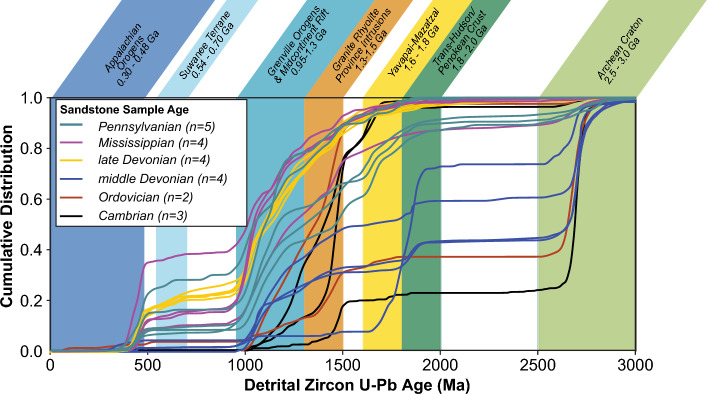
Cumulative density plot of U–Pb detrital zircon ages from Paleozoic strata in the Michigan Basin including 18 new samples from this study and 5 samples published in Thomas et al. (2020). Contemporaneous samples in Cambrian, Ordovician, and middle Devonian strata show intrabasinal differences in provenance sources whereas late Devonian, Mississippian, and Pennsylvanian samples converge on a common provenance signature.

**Figure 3:**
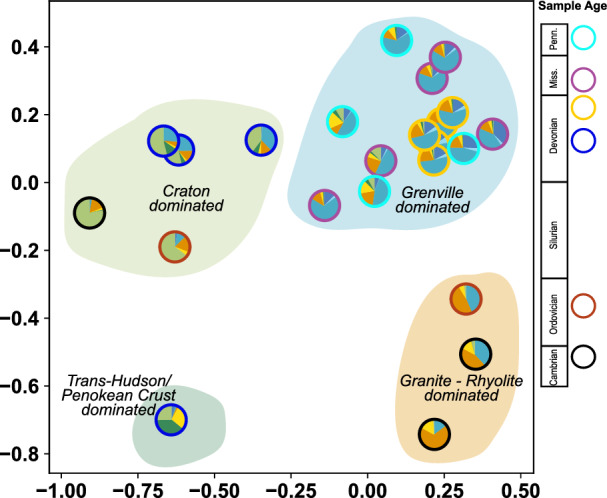
Multidimensional scaling plot of detrital zircon distributions from Paleozoic strata in the Michigan Basin including 18 new samples from this work and 5 analyses from Thomas et al. (2020). Individual samples are represented as a single pie graph. Pie graph colors reference source terranes in Fig. 1. Four distinct detrital zircon signatures are recognized in Paleozoic strata of the Michigan Basin and identified by the fields in the MDS plot.

U–Pb analyses were completed for 18 new sandstone samples (collected from cores) from the Michigan basin ranging in age from Middle Cambrian to Lower Pennsylvanian (Fig. 2; Supplementary Information). Middle Cambrian to Lower Pennsylvanian detrital zircon samples from the Michigan Basin can be categorized according to the dominant U–Pb ages associated with a specific primary source terrain although most categories exhibit consistent proportions of U–Pb ages from lesser zircon age components as well (Fig. 3). Using this classification scheme we observe four distinct signatures: (1) *Craton dominated samples* are characterized by > 50% of U–Pb ages 3.0–2.5 Ga and lesser zircon age components of 1.3–0.95 Ga, 1.5–1.3 Ga, 2.0–1.8 Ga, and 1.8–1.6 Ga grains; (2) *Granite rhyolite dominated sample*s with > 40% of U–Pb ages 1.5–1.3 Ga and lesser zircon age components of 1.3–0.95 Ga and 1.8–1.6 Ga with notably few grains 3.0–2.5 Ga; (3) *Trans-Hudson/Penokean dominated samples* with > 40% of U–Pb ages 2.0–1.8 Ga and lesser zircon age components of 1.8–1.6 Ga and > 2.5 Ga grains; (4) *Grenville dominated samples* with > 50% of U–Pb ages 1.3–0.95 Ga and lesser zircon age components of 1.5–1.3 Ga, 0.48–0.3 Ga, 1.8–1.6 Ga, and 3.0–2.5 Ga grains. These signatures are represented in multi-dimensional scaling (MDS) maps of the samples from the Michigan Basin (this study and Thomas et al., 2020) that provide a visual comparison of the statistical similarity of samples (Fig. 3).

**Figure 4:**
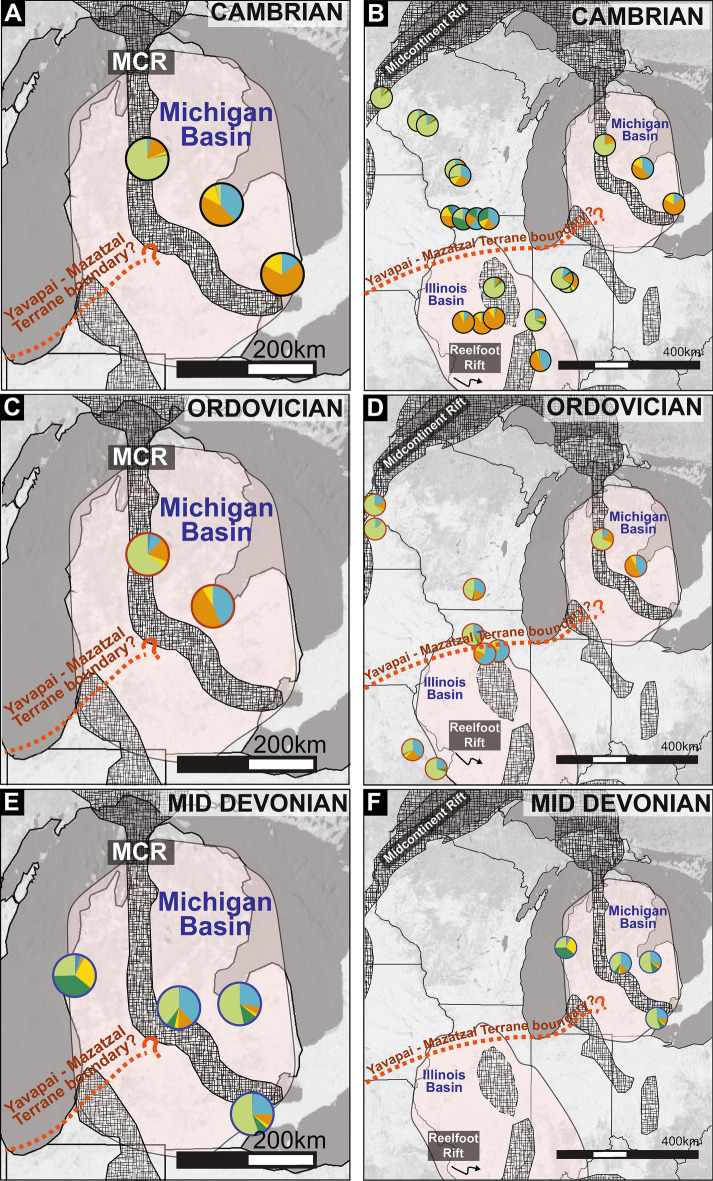

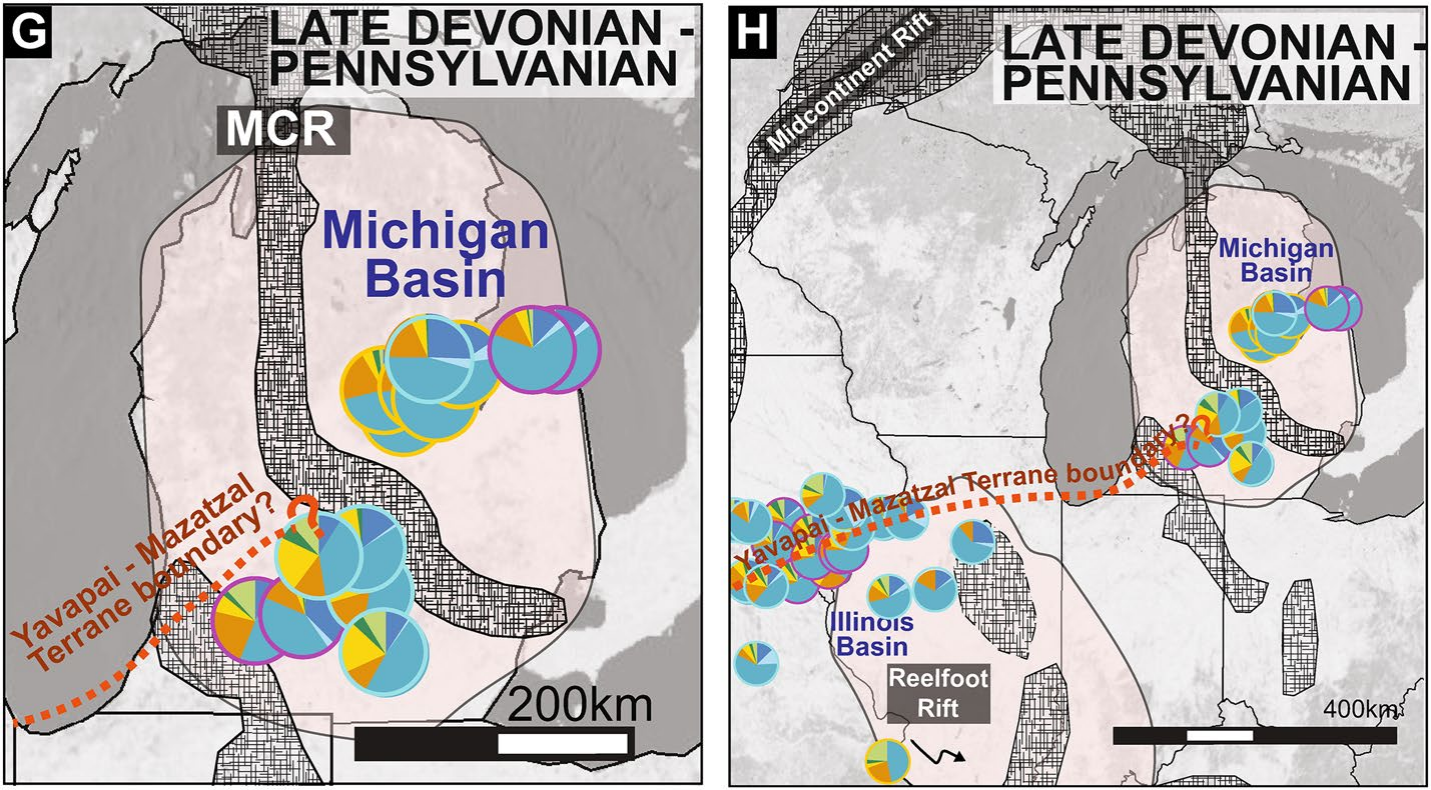
Regional maps of the Michigan Basin (**A**-**D**) and eastern Midcontinent of North America (**E**-**H**) showing new and published detrital zircon data as pie diagrams for four key time periods. Pie graph colors reference source terranes in Fig. 1. Cambrian, Ordovician, and middle Devonian heterogeneity within the Michigan Basin and throughout the eastern Midcontinent demonstrate the important role of cratonic basins in the organization of sediment routing pathways. Location of tectonic features are from Marshak & Paulsen 1996; 1997; Stein et al. 2018; Hinze anf Chandler 2020; Elling et al. 2022. Shaded relief basemap is GEBCO Grayscale Basemap 2021 compiled using ESRI ArcMap v.10.7 under fair terms use (https://www.esri.com/en-us/legal/copyright-trademarks).

The geographic distribution of zircon signatures in samples from Cambrian Mt. Simon Sandstone (n = 3) and Ordovician St. Peter (n = 2) sandstone is the same for both time periods demonstrating sustained sediment source and routing pathways for at least 50 Myrs (Fig. 4A,B). For this time period (Cambrian-Ordovician), Granite-Rhyolite dominated samples on the eastern edge of the basin contain materials originally from basement rocks underlying the basin itself including zircons from the Granite rhyolite and Yavapai-Mazatzal Provinces, and either the Midcontinent Rift or less likely the Grenville orogen to the east (Figs. 1, 4). These zircon age components could be from the primary sources that were eroded along basement-incised margins of the basin or recycled from the Neoproterozoic Jacobsville Sandstone exposed along the reactivated Midcontinent Rift north of the basin^27,38^. Samples from the north-central basin have a craton dominated signature with most material derived from the Archean craton to the north and northwest of the Michigan Basin (Figs. 1, 4). Notably, the major and minor components of Granite-Rhyolite dominated samples and craton dominated samples show very little mixing despite the geographic proximity (< 100 km) of samples with different zircon signatures (Figs. 2, 3). Granite-Rhyolite dominated samples have less than 4% of craton-derived zircons and craton dominated samples have a comparatively minor component (< 25%) of Granite-Rhyolite derived zircons. Granite-Rhyolite dominated samples also have a zircons derived from Midcontinent Rift or Grenville (1.3—0.9 Ga) sources which is nearly absent (< 10%) in craton dominated samples. These “unmixed” signatures support highly localized provenance and/or segmentation within the Michigan Basin in the Cambrian through Ordovician (Fig. 4).

Intrabasin heterogeneity with little evidence for mixing is maintained through the middle Devonian although the primary sources change in parts of the basin (Fig. 4). Samples from the penecontemporaneous Filer (n = 1) and Sylvania (n = 3) sandstones yield two distinct zircon signatures. In the eastern basin, three samples from the Sylvania Sandstone have craton-dominated signatures suggesting that by the Devonian, sediment supply in this area had shifted from local basement or recycled (Jacobsville Sandstone) sources to external sediment supply from the Archean craton (Fig. 4). In the western basin, a sample from the Filer Sandstone has a Trans-Hudson/Penokean dominated signature suggesting significant sediment input from north—northwest of the Michigan Basin either from primary sources or recycled from the Baraboo Quartzite^7^.

By the late Devonian through the Pennsylvanian, all samples in the Michigan Basin including samples from the Upper Devonian Berea Sandstone (n = 5), Lower Mississippian Marshall Sandstone (n = 2), and Lower Pennsylvanian Saginaw Formation (n = 2) yield Grenville dominated signatures suggesting both a shift in sediment source and an integration of sediment routing systems^5^ (Figs. 2, 4).

## Discussion and conclusions

### Regional comparisons of sediment mixing

A regional compilation of detrital zircon data throughout the eastern Midcontinent illustrates the existence of localized depocenters both within and between cratonic basins during the Cambrian to middle Devonian^3,15,39–41^ (Fig. 4E–H). Many of these depocenters are bounded by inherited or reactivated tectonic features. For example, Cambrian strata with abundant 1.5–1.3 Ga (Granite-Rhyolite) zircons are concentrated along the margins of the Reelfoot Rift/LaSalle Deformation Belt with minimal apparent mixing with other depocenters that are dominated by 3.0–2.5 Ga (Archean craton) zircons^15,39–41^. During the Ordovician, some depocenters are dominated by 1.3–0.95 Ga zircons from Grenville Province sources in the east whereas others contain mostly 3.0–2.5 Ga zircons from the Archean craton^3,40,41^. Although data are limited, the variability in the Cambrian—middle Devonian provenance data suggests that sediment was poorly mixed producing local and regional provenance patterns throughout the midcontinent for the early part of the Paleozoic.

New DZ results from latest Devonian show the homogenization of the Michigan Basin by the latest Devonian, although there are limited DZ data (n = 1) beyond the Michigan Basin to evaluate if this homogenization stretched across the midcontinent. From the Mississippian—Pennsylvanian DZ results from strata in the Michigan Basin are consistent with previous DZ analysis from throughout the Midcontinent documenting the initiation of a transcontinental drainage network that promotes mixing within and between sedimentary basins in the Midcontinent of North America during Alleghenian mountain building^3,5,14,41^ (Fig. 4D,H).

### Effective sediment barriers within cratonic basins

The data from this study suggest that provenance heterogeneity within the Michigan Basin was sustained for at least 100 Myrs from the Cambrian through the middle Devonian. These new observations indicate that cratonic basins can sort sediment internally and that subsidence within cratonic basins may serve as an effective barrier to regional and continental sediment routing systems, particularly during periods of plate margin quiescence. However, the mechanism(s) required to facilitate such localized depocenters within cratonic basins—a setting with characteristically little or no topography, little internal deformation, and slow subsidence/shallow accommodation space^8^—is not obvious. We propose possible conditions and mechanisms that may singularly or collectively contribute to this behavior.

Sedimentary processes alone may be sufficient in some instances to sort sediment and produce variable provenance signatures. Drainage from unique sources along the edges of the oblong basin may produce local deltas that do not mix across the basin particularly under low energy conditions^4^. Mixing could be controlled by biologic processes during the early Paleozoic for example, microbial mats can inhibit mixing and burrowing can facilitate mixing^42,43^. Alternatively, if sediment pulses from unique sources are asynchronous, high-energy conditions and relatively rapid subsidence may preserve tongues of single source sands throughout the basin that reflect event-scale deposits, a phenomenon that is also observed in synorogenic strata of both plate-margin and rift basins^44,45^. None of these conditions were previously interpreted for the Cambrian (Mt. Simon), Ordovician (St. Peter), and middle Devonian (Sylvania, Filer) clastic units sampled from this study for the Michigan Basin. These units are widely interpreted as medium—high energy, well-mixed marine shelf and shoreface deposits^29,46^ in a slowly subsiding basin^8,10^. The two distinct DZ signatures observed in the Cambrian and Ordovician strata are geographically consistent across both time periods suggesting that alternating tongues of sand from unique provenance sources is an unlikely explanation.

The ineffectiveness of sediment mixing may be tied, in part, to sediment supply. During periods of low sediment supply, sediment deposited in an underfilled basin with little to no sediment bypass may be localized around the basin margins even in high energy depositional environments. Because the basin is underfilled, sediment would be locally trapped in the basin minimizing mixing. During periods of high sediment supply, an overfilled basin could facilitate sediment bypass through the basin promoting mixing within the basin and beyond its edges that dwarfs localized provenance signatures. High sediment supply at plate margins could bury both local sediment sources and fill the basin further supporting sediment bypass. These scenarios are consistent with the timing of the transition from heterogeneous, spatially variable sediment to homogeneous, mixed sediment in the Michigan Basin by the late Devonian following the decay of the Acadian orogeny and the initiation of the Alleghenian orogeny. Homogenization in the Michigan Basin occurs during a period of drainage organization that marks the emergence of continentally-integrated sediment routing systems crossing North America including the Michigan, Illinois, and Forrest City Basins^3,5,47^. These transcontinental drainage systems persist through the Mesozoic suggesting major plate boundary orogens can overwhelm the sediment supply during periods of active mountain building and during the subsequent tens to hundreds of millions of years of orogenic decay^2,3,5,7,14,48,49^.

It is possible that sedimentary processes act in concert with physical topography to promote localized sediment organization in the Michigan Basin. Underlying the Michigan Basin are inherited Mesoproterozoic and Neoproterozoic tectonic features that may be associated with topography that impeded sediment transport. The terrane boundary between the Yavapai and Mazatzal provinces trends east–west through the Michigan basin, although the exact latitude of this boundary is poorly constrained^23,50^ (Fig. 1). The eastern arm of the Midcontinent Rift that initially formed in the Mesoproterozoic, inverted sometime during the earliest Neoproterozoic to earliest Ediacaran^24–26,51–53^ and trends north–south through the basin^54^. Inherited relief or Paleozoic tectonic reactivation^28,53^ along either of these features could produce a physical barrier consistent with the provenance patterns observed in the Cambrian—middle Devonian stratigraphy (Fig. 4). Local exposures of basement rock along these features could also serve as a primary sediment source for the Granite-Rhyolite dominated samples observed in the Cambrian and Ordovician. Published Paleozoic isopach maps and subsidence analysis show shifting depocenters throughout the Paleozoic^22^. Likewise, carbonate stratigraphy documents an Ordovician—Silurian north- south facies divide that may suggest there was some intrabasinal relief during the Paleozoic^55–57^. However, there is limited evidence for tectonically reactivated or inherited topographic relief (> 50 m scale) that bisects the Michigan Basin either N-S or E-W in isopach maps of early Paleozoic strata^10,15,22,32^. Reconciliation of the observed provenance data sets which require intrabasinal sediment divides with isopach maps^15,22,31,32^ suggests that intrabasinal topographic relief, if it existed, was minimal (10 s of meters maximum) relative to overall subsidence.

We prefer a model for sediment mixing in the Michigan basin in which sedimentary processes deposit sediment locally during periods of low sediment supply producing a variable provenance pattern. It is possible that this sediment organization may be impacted by low relief topography, perhaps a long wavelength divide of only 10–20 m in elevation difference, along inherited tectonic features. Importantly, this model calls into question the idea that sheet sandstones represent the end product of exceptionally long periods (> 10 m.y.) of continental denudation and widespread mixing which would require largely homogeneous provenance signatures over local and regional scales^17–19^. Instead, our study demonstrates that sheet sandstones can have variable provenance from local sources with minimal mixing.

### The effect of cratonic basins on continental-scale sediment routing systems

The persistence of poor sediment mixing within the Michigan Basin for over 100 Myrs in the first half of the Paleozoic suggests that cratonic basins exert an important control on sediment transport pathways. During periods of low sediment supply typical of plate margin quiescence, underfilled cratonic basins may be capable of storing all locally and regionally derived sediment causing cratonic basins to serve as effective sediment barriers. In the center of the Michigan Basin, > 3 km of Cambrian—Devonian strata (pre-Alleghenian) preserved poorly mixed zircon signatures that support this idea. During periods of high sediment supply, for example during plate margin orogenesis, subsidence in cratonic basins may be insufficient to accommodate all sediment and sediment bypass through filled cratonic basins drives transcontinental sediment routing systems. The upper Paleozoic strata of the Michigan Basin have similar zircon signatures that support increased mixing associated with sediment bypass. Although it is possible that the Michigan Basin represents an anomaly of early Paleozoic sediment routing patterns in the US Midcontinent, a regional survey compiled from previously published data sets suggests that localized sediment routing systems may have been common throughout the continent prior to Alleghenian mountain building^3,5,14–16,40^. This is also consistent with observations of localized sedimentation patterns in Cambrian strata of the southwestern US along low-relief landforms following the Great Unconformity^58^. These examples demonstrate that cratonic basins, perhaps in conjunction with low sediment supply and/or internal inherited low-relief topography, can inhibit the integration of larger regional or continental-scale drainage networks like those observed in the North America during the late Paleozoic^3,5^ and the Cretaceous^49^ producing localized depositional patterns and making it more difficult to predict the redistribution of clastic materials via sediment transport pathways using regional studies with samples separated by as little as 100 km.

## Methods

### Detrital zircon U–Pb geochronology

Whole rock samples were collected from cores archived at the Michigan Geological Repository for Research and Education. Zircons were extracted using standard crushing and sieving followed by magnetic and density separation methods. Grains were mounted in epoxy, polished to expose grain interiors, and backscatter electron (BSE) and/or cathodoluminescence (CL) images were generated for each sample at the University of Arizona prior to analysis. U–Pb analyses were conducted at the University of Arizona’s Laserchron Center using laser-ablation inductively coupled plasma mass spectrometry (LA-ICP-MS). Up to 300 grain cores were targeted for each sample with fewer grains analyzed for samples with low zircon yields. We report the ^206^Pb/^207^Pb ages for grains older than 1.0 Ga and the ^206^Pb/^238^U age for grains younger than 1.0 Ga.

### Multidimensional scaling analysis

Multidimensional scaling (MDS) is a statistical tool that allows us to evaluate the similarity of U–Pb age distributions across many samples by plotting samples on a map using a 2D coordinate system. Samples that plot closer in an MDS map are more alike than samples that plot farther apart. We used detritalPy^59^ to produce an MDS map for all new (n = 18, this study) and published^5^ (n = 5) Middle Cambrian to Lower Pennsylvanian detrital zircon samples from the Michigan Basin. MDS calculations used the total of maximum differences between two samples’ cumulative density functions (Kuiper V_max_). This approach evaluates if two samples are derived from the same distribution and is sensitive to all age populations within a distribution^60,61^. MDS calculations for samples from the Michigan Basin (this study and Thomas et al. 2020) have a stress value of 0.6004 (Supplementary Information).

## Supplementary Information

Supplementary Information.

## Data Availability

New DZ datasets from this study have been uploaded to Geochron.org.
